# Editorial: Mechanobiology of organoid systems

**DOI:** 10.3389/fcell.2024.1369713

**Published:** 2024-01-30

**Authors:** Shijie He, Claudia Tanja Mierke, Yubing Sun, Jeroen Eyckmans, Ming Guo

**Affiliations:** ^1^ Clinical and Translational Epidemiology Unit, Department of Medicine, Harvard Medical School and Massachusetts General Hospital, Boston, MA, United States; ^2^ Faculty of Physics and Earth Science, Peter Debye Institute of Soft Matter Physics, Biological Physics Division, Leipzig University, Leipzig, Germany; ^3^ Department of Mechanical and Industrial Engineering, University of Massachusetts Amherst, Amherst, MA, United States; ^4^ Kilachand Center for Life Sciences and Engineering, Department of Biomedical Engineering, Boston University, Boston, MA, United States; ^5^ Department of Mechanical Engineering, Massachusetts Institute of Technology, Cambridge, MA, United States

**Keywords:** mechanobiology, organoid, lung stiffening, senescence, 3D force quantification

## Mechanobiology and diseases

Seminal studies in the mid 2000s reported that extracellular matrix (ECM) stiffness (Engler et al., 2006), cell shape and cellular contractility (McBeath et al., 2004) can direct mesenchymal stem cell differentiation. During the subsequent 20 years, the field of mechanobiology has been significant inspired and substantially developed. These studies helped to better understand how mechanical forces regulate complex cell behaviors and tissue functions (Han et al., 2018; Li et al., 2021a; Li et al., 2021b), and influence homeostasis and disease development (Chowdhury et al., 2021).

Mechanical forces play a pivotal role in regulating cellular biochemical signaling pathways, with reciprocal interactions influencing both cellular activities and mechanical properties in response to environmental cues (Han et al., 2018; Yang et al., 2023). These mutual interactions between mechanical forces and biochemical signaling pathways are critical to human health and disease development. In general, stiffening of the ECM during inflammatory diseases, fibrotic diseases or tumor development can regulate cellular signaling pathways, such as YAP/TAZ, via increasing cellular tractions on ECM, contributing to pathogenesis and exacerbating disease outcomes (Ingber, 2003; He et al., 2022; He et al., 2023). In the realm of glaucoma research, Du et al. uncovered a role for cellular senescence in disrupting the mechanoresponses of trabecular mesh cells (TMCs). Senescent TMCs, subjected to fluid shear stress, exhibited diminished F-actin formation, poor realignment of F-actin fibers, reduced cellular stiffness, and abnormal expression of ECM remodeling-related genes, compared to their non-senescent counterparts. In another study by Chi et al., deficiency in Integrin β4 expression led to increased lung tissue stiffness and elevated ECM components, such as collagen and elastin. Furthermore, Integrin β4 deficiency hindered the adaptation of bronchial epithelial cells to the ECM stiffening due to decreased cytoskeletal stabilization and impaired RhoA activity, ultimately contributing to the development of lung dysplasia.

In addition to affecting cytoskeletal proteins, mechanical forces can activate mechanosensitive Piezo proteins, which serve as pore-forming subunits of ion channels at the cell membrane. In response to mechanical stimuli, such as pressure, shear, and stretch, Piezo ion channels open and allow positively charged ions to flow into the cell, including calcium (Wu et al., 2017). As reviewed by George and Bates in this Research Topic, calcium oscillations occur in almost all cell types and tissues, playing a crucial role in morphogenesis and tissue development. Disruptions of these oscillations can lead to developmental abnormalities and pathogenesis. However, the underlying mechanisms by which mechanical stimuli impact bioelectrical signals and associated pathophysiological functions remains unclear.

## Mechanobiology in organoid systems

The role of mechanical forces in cell proliferation, differentiation, and migration have been extensively studied (He et al., 2014; He et al., 2015; Guo et al., 2017; He et al., 2019; He et al., 2023). However, due to the complexity of living organisms, it is challenging to interpret how these mechano-biochemical coupling signaling pathways impact complex organ-level functions. The emerging technique of organoid culture provides a feasible platform recapitulating *in vivo* organ anatomy and functions for researchers to connect the cellular level mechanisms with the organ-level behaviors, including organoids of brain, lung, kidney, and gut, among others. In this Research Topic, Nauryzgaliyeva et al. presented a comprehensive reviews wherein they introduced the cutting-edge human pluripotent stem cells (hPSCs)- kidney organoid culture which faithfully captures *in vivo* kidney development and diseases. As they pointed out, the mechanical cues have largely been unexplored within hPSCs-derived organoid cultures. These studies in the future will help to better understand their impact on organ development and disease pathogenesis. They comprehensively reviewed the state-of-the-art techniques to interrogate organoid mechanobiology, including mimicking the extraembryonic microenvironment, using natural or synthetic substrates, combining with microfluidic devices, manipulating mechanosensing and mechanotransduction machineries, and measuring forces in complex organoids.

Regarding quantification of mechanical forces in 3D system, like organoids, Tian et al. reported a novel strategy in this Research Topic to analyze E-cadherin mediated intercellular forces using a series of DNA-hairpin molecular probes which they have developed for 2D cell models (Zhao et al., 2017; Zhao et al., 2020; Kes et al., 2021). Excitingly, after 1–2 h of incubation, these small molecule probes can penetrate a dosage-dependent depth of 50–200 µm of various 3D spheroids, including embryonic stem cell-derived embryoid bodies with strong cell-cell junctions. Combined with confocal microscopy or potentially more advanced imaging tools such as light sheet microscopy, this advanced technology will facilitate the quantification of complex intercellular mechanical interactions within 3D organoids.

## Organoids and mechanomedicine

Throughout daily life, cells, composing living organisms, experience various mechanical forces, such as stretch, shear and pressure, as well as encounter different material properties, including varying stiffness, viscosity, surface roughness, and geometries. These constitutive/inherent mechanical cues can regulate cellular signaling pathways and reshape functions of muscles, bones, heart, and other organs, ultimately impacting human health as aforementioned. Targeting mechanosensing pathways is indispensable to tackle diseases and improve human health. Organoid-based systems bridge the cellular level signaling pathways with organ level functions in basic research and clinical studies, which are guaranteed to provide a powerful system for the fields of mechanobiology and mechanomedicine.
